# Targets’ Coping Responses to Workplace Bullying with Moderating Role of Perceived Organizational Tolerance: A Two-Phased Study of Faculty in Higher Education Institutions

**DOI:** 10.3390/ijerph20021083

**Published:** 2023-01-07

**Authors:** Levia, Gurvinder Kaur

**Affiliations:** School of Humanities and Social Sciences, Thapar Institute of Engineering and Technology, Patiala 147001, India

**Keywords:** workplace bullying, EVLN, coping, perceived organizational tolerance, higher education

## Abstract

This study aims to examine the relationship between workplace bullying and Exit-Voice-Loyalty-Neglect (EVLN) coping responses with Perceived Organizational Tolerance (POT) of bullying as moderator using the integrated model of reactance and learned helplessness theory. The data has been collected from junior faculty in higher education institutes of Punjab. The study has been conducted in two phases, with phase 1 including identifying targets of workplace bullying using cut-off scores and phase 2 studying the perceived organizational tolerance. The results show that junior faculty chooses neglect as a coping response and that the level of perceived organizational tolerance moderates the relationship between workplace bullying and exit-voice-neglect. This study contributes to existing literature by employing integration of theories and using multi-level research design. It also is an addition to the literature on the coping response of targets to workplace bullying in higher education and is a rare attempt at operationalizing perceived organizational tolerance and its relation with workplace bullying.

## 1. Introduction

Free unconstrained thoughts, important for the enhancement of knowledge and the verve of a self-ruled society, emerge from the places known as universities [1]. The universities provide knowledge promoters with academic freedom, by supporting their expression of ideas, indifferently, whether they are controversial, fictitious, uncommon, or may attract retaliation [2]. This very aspect, that makes our higher education system irreplaceable, attracts numerous challenges from various routes. Meeting global standards, unsettling technologies, funding concerns, knowledge modification, areas of obsolescence, and marketization create enormous pressure on the institutions to survive, on faculty to deliver and on scholars to cope [2]. Such internal-external stressors, themselves sometimes categorized as bullying [3] make it difficult for the universities to survive in the resource-constricting environment and pose challenges for the faculty with their constant changing roles [4].

Workplace bullying, the obstruction to decent work [5], has been documented as a global concern [6]. Elimination of violence and harassment from workplaces marked as a significant goal with the adoption of Convention 190 by the International Labour Organization in 2019 [7]. The progress of Asian nations towards the achievement of sustainability, the blueprint of United Nation’s Sustainable Development Goals [8], can be mapped with the achievement of peace and prosperity throughout the region and making the functional institutions stronger [9].

Bullying in academe is perceived as wide-spread with universities being amongst the worst workplaces [2,10]. Further, examining the impact of bullying in academia, it is affirmative that losses occurs at a number of levels: loss for the one who is victim to bullying (health and wellbeing); the cost to the department (loss of an employee, upset schedules); damage for students (reduced teaching quality and unsatisfying mentoring); loss to institutions (of time, money and resources); and failure for society (undermined intellect and freedom) [2,11,12].

Research on workplace bullying in the education sector primarily originates from western countries such as USA [13], Canada [13], Europe [14], Australia [15] and New Zealand [16]. Further, increasing research from the other parts of the world such as the Middle East [17], Africa [18], India [19] and Pakistan [12] can also be witnessed. Given the significant role of the faculty in academia, the focus of research has been largely on experiences of faculty as targets, witnesses, and actors in workplace bullying [2].

As the educational setting involves faculty, who are mentoring students and impacting their lives, the cost of work stress can be upsetting [20]. The earlier studies have reported that employees who are young, novice, less experienced, relatively have lower level of education, junior faculty and faculty-in-training are more vulnerable to bullying in higher education institutions [2,4,11,12].

Over three decades the research has progressed to study antecedents and outcomes of bullying; however, research relating to coping has emerged noticeably only in recent years [21]. Coping has been defined as the efforts made to tolerate, master or reduce the effects of conflicts [22]. Raising one’s ‘voice’, complaining, support seeking, patience, aggression, problem avoidance, withdrawal from work, intent to leave, quitting the organization, social isolation, etc., are various coping strategies used by the targets to workplace bullying [21].

Of the various coping responses this study employs the framework of exit, voice, loyalty and neglect (EVLN) outcomes, which has been widely accepted [21]. The framework of EVLN to study employee responses in adverse situations was first proposed by Hirschman [23] as the exit-voice-loyalty model of dissatisfaction and later was expanded by others [24,25] introducing neglect as the fourth response.

Workplace bullying, embraced as systematic flaws in the organizations [26] if there are permissive environments to such behaviors, imposes severe threats to their functioning [27]. High tolerance has been described as allowing certain behaviours even though they are considered illegitimate [28]. The question of high organizational tolerance to such negative acts has been raised time to time, yet remains unanswered operationally to date [28].

In academe particularly, the state of job insecurity and dependence on the senior permanent faculty put junior faculty at higher risk and decrease their tendency to defend themselves (‘voice’) [29]. Talking to friends and family [30,31,32] and seeking support from colleagues [33,34] have been frequent responses to the negative experience of workplace bullying. However, friends and family could only provide emotional support, not being from the university [35], and support from colleagues came with the expectation to involve in direct action [34]; informing the significant people in the university has the utmost opportunity of bringing required change [2,36], but given the literature, unfortunately, involvement in the formal complaints system is one of the least chosen responses to workplace bullying [31,33].

Though the research over the decades has supported better understanding of coping responses to workplace bullying, several research gaps can be identified that have been neglected. First, substantial research has focused and amassed evidence based on the resource-depletion and social-relational perspective [37,38,39]. Other theoretical standpoints such as social-influence, self-regulation, routine-activities theory or the integration of different theoretical models seem deserted. Secondly, most of the literature focuses on cross-sectional research design. More prospective and robust designs can be experimented with [38]. Third, very few studies can be identified signifying the importance of organizational moderators of bullying [40]. This aspect is as yet at an emerging stage of the research agenda and has larger scope [38]. Lastly, coping perspectives in the wider education sector and understanding of the organizational perspectives are yet underexplored.

Therefore, this study aims to fill the listed gaps by employing an integrated theoretical model of reactance and learned helplessness theory with the perceived organizational tolerance as the moderator between workplace bullying and the coping responses, focusing only on targets of workplace bullying. The paper follows the course of providing a theoretical background and hypotheses development, research methodology depicting results, followed by discussion and implications, understanding limitation, and discovering future research directions along with conclusions.

## 2. Theoretical Background and Hypotheses Development

### 2.1. Integrated Model of Reactance Theory and Theory of Learned Helplessness

Theory of Psychological Reactance: Brehm [41,42] proposed that when an individual’s behavioral freedom is endangered, the person gets motivated and tries to restore the freedom.

Theory of Learned Helplessness: Seligman and Maier [43] instigated that when a person perceives that the situation is uncontrollable, he or she acquires the sense of helplessness causing motivational, cognitive and emotional deficit.

Integrated Model: Wortman and Brehm [44] proposed an integration based on expectation of control and importance of the outcome to be controlled in order to understand the response of an individual. The integration states that when a person expects to be able to control the situation and finds it important, psychological reactance to the situation arises where an attempt is made to restore the threatened freedom. Further, over a period of time, when the individual, despite efforts, learns that the situation is uncontrollable and does not produce desired results, a state of helplessness is acquired and the individual stops trying. Clearly, reactance precedes helplessness and therefore depending upon the controllability expected and the importance of the same, an individual responds to the situation.

Since an individual employs range of coping strategies in response to negative acts at the workplace [45,46], an integrated model can provide a clear explanation of the same. The following figure depicts employment of the integrated model with EVLN outcomes, where individuals respond in different ways depending upon the motivation to control and exposure to uncontrollable situations.

**Figure 1:**
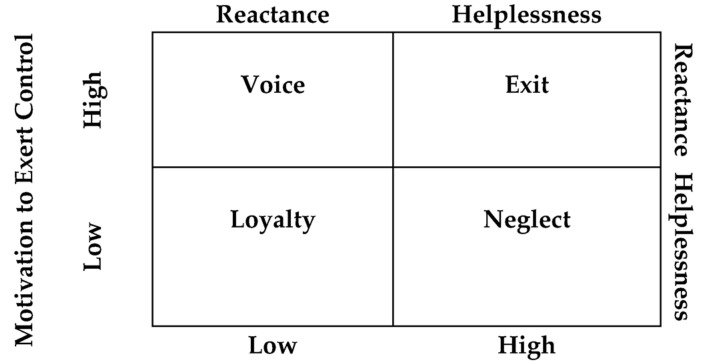
Integration of theoretical model to coping responses.

As per Withey and Cooper [47], when a person has believed that he/she can control the situation but now considers that an improvement in the situation is not likely, the individual tends to exit on exposure to external forces. Therefore, on combining the concept with an integrated model [44] it can be understood that when individuals experience motivation to exert control but are highly exposed to uncontrollable situations exit is practiced. Further when improvement is considered highly likely and expected while controlling the events is considered important, the voice is raised; thus, with high motivation to exert control and less exposure to uncontrollable situations, raising of the voice can be expected. As the exposure to uncontrollability increases, but the motivation to control is high, the individual shifts from voice to exit. In addition to this, Withey and Cooper [47] further concluded that loyalty increases when improvement is less expected and less control on the situation is perceived; therefore, with low motivation to exert control and on exposure to uncontrollable outcomes, the individual tends to practice loyalty. Lastly, when the integrated model [44] is understood in line with the Withey and Cooper [47] predictions, it can be comprehended that when improvement in situation is least expected, motivation to exert control drops and therefore on high exposure to uncontrollable situations, a neglect coping response is employed. As shown in Figure 1, when the motivation to exert control is low and the individual experiences low to high uncontrollability, the coping response tend to move from loyalty to neglect. Thus, Figure 1 summarizes the hypothesized framework through theoretical integration depicting that when motivation to exert control is high, individuals tend to respond actively (exit and voice) using psychological reactance, and when helplessness emerges over the perception of uncontrollability, passive coping responses of loyalty and neglect are sought.

#### 2.1.1. Exit

Exit has been defined as leaving or transferring to a new job or a mere intent to quit [25]. Withey and Cooper [47] state that a person decides to exit when the cost of quitting is low in comparison to raising the voice and when the improvement in the situation is not likely expected. As per the integrated model of reactance and learned helplessness theory [44], on finding themselves in uncontrollable circumstances, the targets experience the stage of helplessness which produces motivational and cognitive deficit, making the individuals believe that under no circumstances can fruitful results be obtained; however, they still hold the motivation to take control of the situation and therefore it is hypothesized that with the exposure to workplace bullying, perceptions of uncontrollability increase and targets tend to quit. According to Zapf and Gross [48], bullying at an advanced stage becomes a non-control event for the targets. This uncontrollability produces helplessness, where quitting the organization emerges as the only solution [48]. Earlier researchers have stated that bullying creates situations at work (unfairness, loss of dignity, etc.) that are difficult for the target to cope with and thus the target tends to choose to quit the organization [26,49,50]. It has been found that when the bullying continues over a period of time and remains unresolved, the exit option is acclaimed [51]. D’ Cruz and Noronha [52] reported in their study that the victims of bullying when deciding to quit are left with mixed feelings. There is a sense of relief for the new beginning; however, the helplessness of not fighting injustice successfully may overwhelm them. Further when the targets were asked about the strategy that one should use when in their situation, unfortunately, exit was recommended as the most effective coping response [48,53,54]. Thus, it has been hypothesized that:

**H1:**  *Experience of Workplace Bullying is positively associated with Exit.*

#### 2.1.2. Voice

Voice has been defined as active and voluntary expression of improving current unwanted situations, discussing challenge with the authorities, conveying suggestions, seeking support or whistle-blowing [25,55]. The belief that raising voice would give effective results and the organization/supervisor/management seems to be supportive is prerequisite for employees to engage in voice; otherwise, silence is preferred [49]. According to the integrated model of reactance and learned helplessness theory [44], when an individual expects that the situation can be controlled and handled and is motivated for the same, the voice option is sought. However, on experiencing workplace bullying over a period of time, exposure to uncontrollability is perceived [48], and therefore, the motivation to restore freedom is lost. The literature also suggests that the one who speaks is considered as a troublemaker, and is further vulnerable to negative impacts [56] of raising one’s voice such as social isolation and loss in professional opportunities, further leading to more workplace bullying [49,57,58]. A few studies have also identified that expression of voice can improve the individual’s status and imply that the person is willing to speak on behalf of others and self [58,59], but such a premise is scarce. Since we deal with the junior faculty and owing to their position as lower-level employees [2], raising voice under helplessness is not preferred, as expectation of the outcome is low. Therefore, it is hypothesized that:

**H2:**  *Experience of Workplace Bullying is negatively associated with raising Voice.*

#### 2.1.3. Loyalty

Loyalty has been described as a passive response of showing optimism which involves waiting for the situation to improve or get better depicting trust in the organization [25]. The employees are loyal to their superiors or the organizations when they have sufficient trust that they will be supported and when they feel safe physically, emotionally and socially [60]. In the study of Withey and Cooper [47], loyalty has been reflected as the feeling of entrapment amongst employees, more than the adherence of support to the organization. Similar results can be seen in the study of Dolev et al., [61] where loyalty has been based on two patterns that is, acceptance and tolerance. As per the integrated model [44], when the motivation to control the outcomes is low and also the exposure to uncontrollable situations (workplace bullying here) is low, loyalty shall be the response. Therefore, when workplace bullying, perceived as an uncontrollable situation, is experienced by the targets, a negative relation with their loyalty can be hypothesized. Further, Mousa [62] in his study found that as the workplace bullying increases with time, the loyalty towards the organization decreases. The employees are less concerned when the bullying affects their quality of personal or professional life and are more concerned when bullying affects health, reputation and social relations; with the occurrence of the latter the statistics of employee loyalty drops [62]. In a study by Leach [63], it was found that the new employees, who were the least experienced teachers, were the ones who showed most loyalty. When employees undergo adverse situations, the sense of loyalty tends to fade away [64] and hence, a negative relation has been established between the workplace bullying and loyalty in the earlier studies [49]. Based on the existing literature following has been hypothesized:

**H3:**  *Experience of Workplace Bullying is negatively associated with Loyalty.*

#### 2.1.4. Neglect

Neglect can be regarded as the negative behavior performed when workplace stressors are perceived [65]. It has been described as passive behavior that allows conditions to get worse by reducing the target’s interest and effort and by increasing the error rate, absences, lateness, etc. [25]. According to D’Cruz and Noronha [66], targets’ persistent feeling of powerlessness and defenselessness creates a negative state. This state of negativity may tend to disrupt emotions and thus employees engage in neglect behavior perceiving the situation as uncontrollable. According to the theoretical framework by Wortman and Brehm [44], targets’ least expecting the change in the circumstance and highly perceiving themselves under the situation of uncontrollability tend to indulge in avoidance coping response [67]. Also, neglect can be the result of protecting personal resources and reducing psychological strain [68]. Dolev et al. [61] found that reduction in the effectiveness of work and revenge intentions were two prominent neglect reactions witnessed of which former was more frequent. Likewise, when Akhmad et al. [69] clustered the employee reactions to negative situations, neglect emerged as a dominant cluster representing employees who would stay with the organization but show withdrawal behaviors of being sick, coming late or employing the least effort in work. Workplace bullying also depicted a positive relation with neglect among Indian managers [49]. Therefore, it has been hypothesized that:

**H4:**  *Experience of Workplace Bullying is positively associated with Neglect.*

### 2.2. Role of Perceived Organizational Tolerance

Organizational tolerance is defined as deliberate or unintentional permissiveness related to negative acts that can take place in an organization. It sometimes further gets extended to collusion, where the related person not only allows but also encourages such acts [28]. Therefore, the organization emerges as the key factor while dealing with counterproductive behaviors such as harassment, bullying, mobbing, etc. [70].

Researchers believe that targets’ perception of tolerance (POT) of workplace bullying in an organization has the ability to alter their perceptions regarding the controllability and uncontrollability of the situation; therefore, perceived organizational tolerance should be tested as a moderator. Therefore, based on the integrated model of reactance and learned helplessness theory by Wortman and Brehm [44], when POT is low, high expectancy and motivation can be anticipated towards engaging in reactance responses and efforts to restore behavioral freedom, whereas when POT is perceived as high, the bullying targets may tend to experience strong feelings of helplessness and thus either respond through exit or neglect.

Cortina [71] found that the main predictor of harassment was organizational tolerance. Supporting the same, Einarsen [72] suggested that a high level of tolerance acts as a precursor to harassment. Following this, Feijó et al. [27] stated that organization’s attitude shapes the work environment which further has a significant relationship with the negative behavior of harassment. Lee [73] studied the effect of organizational tolerance on how often sexual harassment occurs. Further, Hunt et al. [74] found that sexual harassment is more liable to happen where tolerance is perceived high. Einarsen et al. [75] conceptualized the framework for bullying. They stated that organizational actions which include organizational tolerance moderate the effects of bullying on individuals as well as the organization. They depict that the stigmatization process may get altered with the change in the perceptions of victims about how organizations tolerate such behavioral occurrences. Parzefall and Salin [76] emphasize organizational support as a variable that can add to the coping abilities of the employees. It further states that it is possible when the employees perceive that the organizations are supportive to them, there is high chance for the individuals to effectively cope with the negative behaviors occurring at the workplace.

Though most researchers have identified the relation between organizational tolerance and workplace harassment [28], sexual harassment [77,78], workplace incivility [79,80], an workplace aggression [81], little research has been conducted specifying the relation between bullying and tolerance. In the context of workplace bullying, there is ample literature focusing on perceived organizational support [26,76,82,83,84]. The model developed by Einarsen [85] shows organizational tolerance as a part of organizational antecedents and was further used and reframed by Salin [86] in his study; however, creating an operationalized form of the construct has been overlooked. Given the Indian cultural context with the high power-distance and associated high tolerance [87,88,89,90,91], perceived organizational tolerance finds more relevance. Thus, it is hypothesized that:

**H5:**  *Perceived Organizational Tolerance moderates the relationship between workplace bullying and coping responses of EVLN.*

## 3. Research Methodology

### 3.1. Research Design and Sample

The study uses a cross sectional multilevel descriptive design and is based on the junior faculty of higher educational institutions. The study employs a purposive sampling method to collect the data. The focus of the study is to understand the perspective of the targets of workplace bullying and therefore, the respondents who did not qualify as targets were eliminated from the study.

The data collection process was carried out in two phases.

#### 3.1.1. Phase 1

In the first phase the data was collected from the junior faculty (assistant professors) of 17 general universities in the state of Punjab [92], as they form the most vulnerable group to workplace bullying being lower in rank [2,4,12]. A total of 2300 emails with three follow-up reminders at the gap of 3 days each were sent to the assistant professors according to their availability of email ids from the university websites; 774 responses (33.65% response rate) were received [93]. A total of 520 responses were completely filled and hence considered for the study. A further purposive sampling technique was used. These 520 respondents were classified as targets of bullying or not the targets of bullying based on cut-off score of 33. The respondents who scored equal to or above 33 were considered targets [94,95] of workplace bullying and were further approached for the second phase.

#### 3.1.2. Phase 2

In the second phase the items relating to perceived organizational tolerance and coping responses were measured. Of the 520 respondents, a total of 228 respondents were found at par with the eligibility criteria (cut-off scores) and therefore were re-approached to fill the second set of questionnaires; 211 completed responses were received and analyzed.

### 3.2. Measures

The study used following measurement scales to measure the variables of workplace bullying, coping responses (EVLN), and perceived organizational tolerance (POT):

#### 3.2.1. Workplace Bullying

The widely accepted NAQ-R 22 item scale has been used to measure the negative behavior [96]. The scale has been validated in earlier studies and also cut-off scores have been previously calculated to classify the respondents into targets and non-targets of workplace bullying [94,95]. The Cronbach’s Alpha was noted to be 0.891, depicting good reliability [97]. The scale used a 5-point timeline ranging from 1 meaning ‘never’ to 5 meaning ‘daily’.

#### 3.2.2. Coping Responses

To measure the coping responses, the EVLN typology by Rusbult et al. [25] has been used. It includes 17 items (4-exit, 4-voice,4-loyalty and 5-neglect). The Cronbach’s Alpha was noted as 0.819 for Exit, 0.811 for voice, 0.673 for loyalty (acceptable as per Ghazali [98]), 0.793 for neglect, and 0.749 overall [97]. The scale was a 5-point Likert scale ranging from 1 ‘strongly disagree’ to 5 ’strongly agree’.

#### 3.2.3. Perceived Organizational Tolerance

To measure the perceived organizational tolerance, the POT scale developed by Perez et al. [28] was modified. The scale measures the POT with respect to harassment, so the items were modified according to bullying context and also those items were considered which were relevant to Indian work culture. The 13-item scale reported the reliability of 0.935 as Cronbach value. The scale was a 5-point Likert scale ranging from 1 ‘strongly disagree’ to 5 ‘strongly agree’.

#### 3.2.4. Control Variables

The demographic variables of gender, age and experience were controlled in the study. Experience of working in the current organization was considered.

### 3.3. Common Method Variance

To address the CMV among the variables [99], Harman’s single factor test was used. Since the first factor accounted for 22.3 per cent of the total variance extracted after factor analysis, there was no concern for CMV, which is of concern only when the first factor accounts for more than 50 percent of the covariance [100].

### 3.4. Data Analysis

The data was analyzed using SPSS 22, Amos 23 and Process Macro 3.5. Moderation analysis (Model 1) [101] was conducted to understand the moderating effect of perceived organizational tolerance between workplace bullying and the coping responses.

## 4. Results

### 4.1. Preliminary Analysis

The measurement model was built using Amos 23 and the model fit was found in the acceptable range [102], as shown in Table 1. Further, the descriptive statistics and the correlations among variables are depicted in Table 2.

#### 4.1.1. Validity of the Constructs

Validity of the scales was measured through convergent and discriminant validity. AVE was measured for convergent validity and discriminant validity was measured based on Fornell and Lacker’s criteria [103] where discriminant validity should be greater than correlation values of the variables of the study. Table 3 shows the CR, AVE, Cronbach Alpha and Discriminant Validity scores depicting validation of the instrument.

#### 4.1.2. Demographic Characteristics of the Respondents

The three demographic variables of gender, age and experience were added to the study. The division of the sample into different categories has been shown in Table 4.

### 4.2. Identfication of Targets of Workplace Bullying

Prevalence of workplace bullying was calculated based on the cut-off scores [94,95,96]. Targets who scored from 33–40 were considered at risk of being bullied [95], 40–56 as occasionally bullied and greater than 56 as severely bullied [94]. The respondents who scored less than 33 were eliminated from the study. Of the total sample, 43.8% of the respondents were classified as targets (228 of 520); further, amongst these 228 respondents, 43.4 percent were classified as at risk of being bullied. In addition, 40.3% were occasionally bullied and 16.3 percent were being severely bullied.

### 4.3. Test of Hypotheses

The targets’ scores were then regressed on coping responses. It was found that of the four EVLN responses, only neglect as a coping response was significantly dependent on workplace bullying (Table 5). This significant relationship establishes that the Indian junior faculty relies more on neglect behaviors than choosing any other coping response. It thus becomes interesting to understand if the organizational intervention brings any necessary change to the targets’ responses, providing a strong reason to test POT moderation. The moderation analysis was conducted using Hayes’ PROCESS MACRO, Model 1. Interestingly, it can be depicted that POT as an independent variable impacted the three coping responses of Exit, Voice and Loyalty, while Workplace Bullying impacted neglect significantly, supporting H_4_ only. With non-significant relationships emerging between Workplace Bullying and Exit-Voice-Loyalty, H_1_, H_2_, H_3_ are not supported significantly. The results of the interaction effect of workplace bullying and perceived organizational tolerance along with control variables are shown in Table 5. On studying the interaction effect of the two it is visible that exit, voice and neglect are the significant outcomes for the interaction, loyalty being not significantly affected, H_5_ being partially supported.

**Figure 2:**
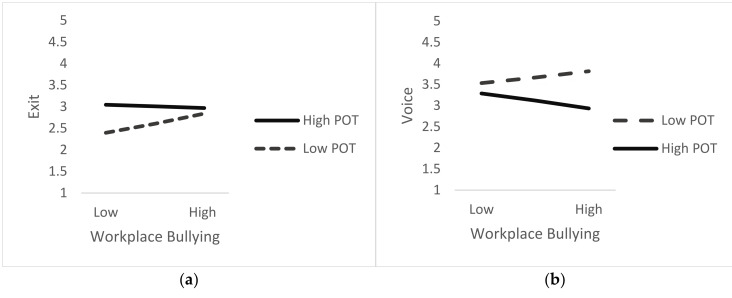

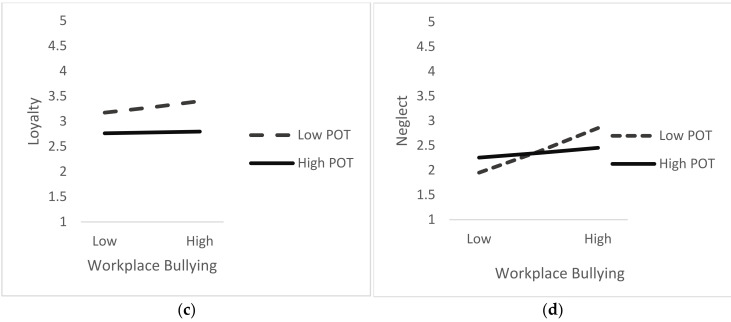
Relationship between workplace bullying and coping responses (EVLN) with POT as moderator. (**a**) Workplace Bullying and Exit; (**b**) Workplace Bullying and Voice; (**c**) Workplace Bullying and Loyalty; (**d**) Workplace Bullying and Neglect.

From the results (Figure 2), it can be interpreted that perceived organizational tolerance of bullying significantly moderates the relationship between workplace bullying and exit-voice-neglect. It can be observed that when POT is low, raising of the voice is preferred. However, at the same time, with a low perception of tolerance, exit and neglect response increase as well. POT does not significantly moderate the workplace bullying and loyalty relation but the low POT does contribute towards increased loyalty, although insignificantly.

On further expanding the conditional effects to +1 SD and −1 SD (Table 6), it was found that the relation of voice with workplace bullying becomes significant when perceived organizational tolerance is low; the relation of neglect with workplace bullying becomes significant when perceived organizational tolerance is high.

## 5. Discussion

As workplace bullying had been labelled as ‘rife’ in higher education [2], the study mainly focused on the junior faculty, to understand how the faculty at lower levels find themselves intimidated and their positions at risk, since supervisor bullying is common and widely practiced [4,11,12]. The junior faculty understudy in phase 1 of our survey can be largely seen at risk of being bullied or being occasionally bullied.

When these targets to workplace bullying were further studied in the second phase, the results reflected a significant relationship between workplace bullying and neglect as a coping response (Table 5). Workplace Bullying showed a positive relationship with Exit, and a negative relationship with Voice as expected [49] but the relationship was insignificant. However, it was surprising to see a positive relationship between Workplace Bullying and Loyalty. A plausible explanation to this positive relation can be attributed to the theoretical underpinning [44], where targets may find supervisor mistreatment as acceptable [89], tending to perceive themselves less exposed to uncontrollable situations that way and therefore waiting for the time to get things right.

Further, the study by Leach [63], states that the employees who were the less experienced teachers, were the ones who showed most loyalty. Since this study deals with the junior faculty, it can be comprehended that the faculty tries to accommodate to the organizational setting and therefore waits for the situation to get better with time. Further, Wu and Wu [104] stated in their study that even when the employees were ignored by HR, they remained loyal to the organization hoping that with time the situation would get resolved. Workplace Bullying and Neglect showed the most significant relationship. It can thus be interpreted that of all the coping responses of EVLN, the assistant professors prefer to adopt neglect behaviors as a shield. Keashly [2], Karatuna [105] and Rai and Agarwal [106] also noted in their studies that when it is difficult for the faculty to leave a bullying situation through internal transfer or to move to a different academe environment altogether or targets have no energy to deal with the situation, the faculty withdraws itself from work in ways that are not productive.

Further when perceived organizational tolerance was allowed to moderate the relationship;, there was an increase in raising of voice when the institution was perceived to have low tolerance for bullying. This signifies the importance of low perceived tolerance, as it induces employees to practise constructive coping strategies [107]. With the low POT, targets tend to get motivated to take control of the situation and therefore get themselves out of the state of helplessness, as stated in the theory [44].

At the same time, it can be witnessed that the faculty who perceived low organizational tolerance also tended to increase their exit and neglect behavior. In the literature, supporting evidence can be seen that with the escalation of workplace bullying, exit and neglect behaviors increase. It is interesting to note that it is more prevalent amongst the employees who perceive that the organization is less tolerant of bullying. This can be understood from the state of learned helplessness [44], where despite the chances to improve the situation, targets have learned that the situation cannot be improved and therefore practice exit-neglect responses. Training to employees where they can get themselves out of uncontrollable situations through plausible solutions can be a possible result oriented strategy to divert the helplessness response of targets to reactance response. Moreover, in a collectivist society like India where there is high preference to belonging to a larger social framework and being rejected or isolated leaves the person rudderless, such coping responses can be comprehended [34,108].

POT does not act as a significant moderator between workplace bullying and Loyalty, but it can be seen that when low tolerance to workplace bullying is perceived, loyalty is high. This shows that the targets tend to increase their loyalty and choose to stick to the organization with the hope of the organization addressing the problem [60].

It was initially expected that low institutional tolerance of bullying would help targets to handle the situation constructively; it is unfortunate to see that low tolerance, while adding to constructive handling, at the same time adds to exit and neglect behaviors as well. These results clearly affirm theoretical propositions [44], that if the actions are taken on time by the organization, it can manage to keep the targets under the reactance bracket of voice and loyalty; however, once the state of helplessness is assumed, it gets difficult to change the perception.

From the previous studies it can be inferred that even in the organizational cultures where tolerance of bullying is perceived as low, the effective action takes a prolonged period of time and the stretched process of dealing with the extended bullying [52,109,110,111,112] forces employees to indulge in behaviors that depict withdrawal. A pre-accepted notion of ‘nothing will be done’ or even if done would make not much difference (leaving them in a ‘status limbo’) in the situation, and rather might bring retaliation, does not allow even the world changers to bring a change in their situation [2].

Therefore, it is recommended for the organizations and the administrations to build a solid foundation by laying down anti-bullying policies and strong implementation of the same so as to ensure that the lag between where a faculty member wishes to raise a voice and needed support is shortened, thereby avoiding the necessity of choosing to exit or practice neglect. A bullying target being loyal to the organization is in some ways a constructive response for the organization; however, is not really constructive, as, if no action is taken, the employee ultimately loses trust in the organization and resolves to shift towards exit or neglect disappointedly [21,105]. The practice of neglect as a coping response not only produces personal or professional cost for the faculty in suffering but at the same time it impacts their ability to engage in mentorship of students, putting their careers, knowledge and course progressions in danger [2].

### 5.1. Implications

#### 5.1.1. Theoretical Implications

The study employs the integrated model of reactance and learned helplessness [44] in terms of coping and workplace bullying. It adds to the literature by employing an integrated model of the two theories, as most of the previous coping literature goes with resource-depletion perspective. Further, the study adopts a multi-level research design by conducting the study in two phases, considering only the targets in the second phase, instead of considering all employees as done by previous researchers at large. This brings a new operationalized perspective on target behavior and adds to the literature of distinguishing between bullied and non-bullied behaviors. Further, to the best of authors’ knowledge, this study is first to use perceived organizational tolerance as an operationalized construct in the literature of workplace bullying and higher education. It also adds to the scarce literature of organizational moderators, to which only a few studies have contributed [40]. In addition, studies in the education sector in Asian countries are scarce and this is an addition to the bullying literature on higher education in Asian nations [2].

#### 5.1.2. Practical Implications

Understanding the coping responses to negative situations at work can empower organizations to form a plan of action. Since low institutional tolerance of bullying provides courage to employees to raise their voice, it is important to build systems such that the targets of any such act can freely reach out for the solution and help. As clearly indicated in the study by Jain [109], there is lack of policy initiative in the Indian organizations, and the few initiatives that exist have indicated patchy results with improper implementation. If such destructive coping response to workplace bullying in higher education continues along with the personal and professional cost to the target, students’ learning experience will be negatively impacted, not forgetting the negative impact that the universities and the administration may bear in terms of reputation and resources. Organizations need to resolve to implement solutions where they can keep up the expectation of employees that the circumstances can be controlled by them, as if they enter the phase of helplessness, turning back becomes a major challenge.

#### 5.1.3. Societal Implications

On the whole, addressing workplace bullying and diverting employees towards the constructive practice of raising their voice, is the social responsibility of every individual. This will empower us towards achievement of the Sustainable Development Goals [8] targeted to be fulfilled by 2030, particularly SDG 3 (Good Health and Well-Being), SDG 4 (Quality and Sustainable Education), SDG 8 (Decent Work), SDG 16 (Strong Institutions). In order to develop a healthy educational system, it is important that participants in this system feel safe and are ready to serve in the best way possible.

#### 5.1.4. Public Health Implications

Workplace bullying negatively impacts the physical and mental health of individuals causing a range of problems such as sadness, anxiety, depression, sleeplessness, decreased physical strength, musculoskeletal disorders and a heightened risk of cardiovascular disease etc. [7]. Therefore, addressing the issue of workplace bullying becomes necessary, to ensure health, safety and wellbeing of the individuals. Better health and wellbeing will further contribute towards increased productivity and effective work performance amongst the faculty of higher education, thereby creating healthy and safe workplaces.

## 6. Conclusions

Workplace bullying in the education sector is common knowledge; however, the response to it is not. Therefore, the study focuses on how the assistant professors working in higher education institutions subject to workplace bullying react to the negative situation. It has been found that they significantly use neglect as coping response. Further, the level of perceived organization tolerance of bullying acts as a moderator between workplace bullying and exit, voice and neglect. The study establishes a base for future studies to better understand tolerance level in higher education organizations to workplace bullying. The results imply that driving improvement in the university workplace will require action taken across the entire system from strategy development to structure, culture and people. Working across all the elements thus can promote the efforts towards finding and implementing solutions.

### Limitations and Future Research Directions

This study follows a cross-sectional design; longitudinal studies can be conducted to understand about the change in coping responses over time. Gender can be considered to understand the varied impact of POT on different genders. Also, tenure of the faculty can be taken into account in the study as the response by permanent and temporary faculty may vary. Further where low perceived organizational tolerance of bullying can induce confidence amongst junior faculty to raise their voice, it can be seen that the mere perception of low tolerance is not enough. Properly laid down policies, assurance of immediate remedy, social support by peers and empowerment of bystanders can play a significant role in curbing destructive coping responses. Based on the results of this study, bystander training [113] can be a strong influencer in allowing targets to respond constructively and raise their voice. Future studies can focus on this aspect of research by empirically testing the proposed explanations and seeking robust findings to yield certain results.

## Figures and Tables

**Table 1:** Model Fit Indices.

Measure	Estimate	Threshold	Interpretation
CMIN/DF	3.869	Between 1 and 5	Acceptable
CFI	0.911	>0.90	Acceptable
GFI	0.913	>0.90	Acceptable
SRMR	0.072	<0.08	Excellent
RMSEA	0.078	<0.08	Acceptable

**Table 2:** Mean, Standard Deviation and Correlation among variables.

Variables	1	2	3	4	5	6	Mean	Std. Dev
**Workplace Bullying**	1						45.65	14.094
**Perceived Organizational Tolerance**	−0.327 **	1					2.76	0.876
**Exit**	0.068	−0.194 **	1				2.76	1.081
**Voice**	−0.237 **	0.327 **	−0.007	1			3.34	0.925
**Loyalty**	0.063	0.305 **	−0.092	0.300 **	1		3.01	0.755
**Neglect**	0.179 **	−0.047	0.317 **	0.094	0.063	1	2.30	0.906

** Correlation significant at *p* < 0.01.

**Table 3:** Validity and reliability of the variables of the study.

Variables	CR	AVE	Cronbach’s Alpha	Discriminant Validity
Workplace Bullying	0.715	0.562	0.867	0.749
POT	0.867	0.571	0.933	0.756
Exit	0.835	0.740	0.795	0.860
Voice	0.790	0.665	0.804	0.816
Loyalty	0.728	0.572	0.733	0.757
Neglect	0.853	0.544	0.799	0.738

**Table 4:** Demographic Characteristics.

Demography	Number of Respondents	Percentage
**Gender**		
Male	70	33.2
Female	141	66.8
**Age**		
20–35	146	69.2
35–50	55	26.1
50–65	10	4.7
**Experience**		
6 months to 1 year	61	28.9
1 year to 5 years	91	43.1
More than 5 years	59	28.0

**Table 5:** Moderating role of Perceived Organizational Tolerance.

Variables and Steps	Exit	Voice	Loyalty	Neglect
**Step1: Controls**				
Gender	−0.119	0.4277 *	0.2043	−0.2357
Age	−0.115	0.0050	0.0786	−0.3002 *
Experience	0.111	−0.0782	−0.0096	0.1562 *
**Step 2: Independent Variables**				
Workplace Bullying	0.006	−0.0014	0.0049	0.0206 *
Perceived Organizational Tolerance	−0.230 *	0.3116 *	0.2870 *	0.0165
**Step 3: Interaction Term**				
Workplace Bullying x Perceived Organizational Tolerance	0.011 *	0.0136 *	0.0042	0.0150 *
**R^2^**	0.0735	0.2179	0.1138	0.1535
**R^2^ Change**	0.0213	0.0432	0.0061	0.0554
**F**	4.6823 *	11.2558 *	1.4019	13.3570 *

Note(s): *n* = 211, * *p* < 0.05.

**Table 6:** Conditional effects of moderation.

Moderator/Effect	Exit	Voice	Neglect
Perceived Organizational Tolerance			
**+1 SD**	−0.0028	−0.0133 *	0.0074
**Mean**	0.0069	−0.0014	0.0206 *
**−1 SD**	0.0166	0.0105	0.0338 *

Note: * Significant at *p* < 0.05; +1 SD depicts low POT and vice versa.

## Data Availability

The data file is available with the author and can be obtained upon a reasonable request.

## Abbreviations

ELVN	Exit-Voice-Loyalty-Neglect
POT	Perceived Organizational Tolerance
AISHE	All India Survey on Higher Education
NAQ-R	Negative Acts Questionnaire-Revised
CMV	Common Method Variance
SPSS	Statistical Package for Social Sciences
CMIN/DF	Minimum Discrepancy/Degrees of Freedom
CFI	Comparative Fit Index
GFI	Goodness of Fit Index
SRMR	Standardized Root Mean Residual
RMSEA	Root Mean Square Error of Approximation
AVE	Average Variance Extracted
CR	Composite Reliability
SD	Standard Deviation
HR	Human Resource
SDG	Sustainable Development Goals
